# Ion Beam Nanostructuring of HgCdTe Ternary Compound

**DOI:** 10.1186/s11671-017-2093-x

**Published:** 2017-05-02

**Authors:** Aleksey B. Smirnov, Rada K. Savkina, Ruslana S. Udovytska, Oleksandr I. Gudymenko, Vasyl P. Kladko, Andrii A. Korchovyi

**Affiliations:** 1 V. Lashkaryov Institute of Semiconductor Physics, National Academy of Sciences of Ukraine, 41 Prospect Nauky, Kyiv, 03028 Ukraine; 2 V. Lashkaryov Institute of Semiconductor Physics, National Academy of Sciences of Ukraine, 45 Prospect Nauky, Kyiv, 03028 Ukraine

**Keywords:** HgCdTe, IR and sub-THz detector, Ion implantation, 61.72.uj, 71.20.Nr, 72.20.Pa

## Abstract

Systematic study of mercury cadmium telluride thin films subjected to the ion beam bombardment was carried out. The evolution of surface morphology of (111) Hg_1 − *x*_Cd_*x*_Te (*x* ~ 0.223) epilayers due to 100 keV B^+^ and Ag^+^ ion irradiation was studied by AFM and SEM methods. X-ray photoelectron spectroscopy and X-ray diffraction methods were used for the investigation of the chemical compound and structural properties of the surface and subsurface region. It was found that in the range of nanoscale, arrays of holes and mounds on Hg_0.777_Cd_0.223_Te (111) surface as well as the polycrystalline Hg_1 − *x*_Cd_*x*_Te cubic phase with alternative compound (*x* ~ 0.20) have been fabricated using 100 keV ion beam irradiation of the basic material. Charge transport investigation with non-stationary impedance spectroscopy method has shown that boron-implanted structures are characterized by capacity-type impedance whereas for silver-implanted structures, an inductive-type impedance (or “negative capacitance”) is observed. A hybrid system, which integrates the nanostructured ternary compound (HgCdTe) with metal-oxide (Ag_2_O) inclusions, was fabricated by Ag^+^ ion bombardment. The sensitivity of such metal-oxide-semiconductor hybrid structure for sub-THz radiation was detected with NEP ~ 4.5 × 10^−8^ W/Hz^1/2^at ν ≈ 140 GHz and 296 K without amplification.

## Background

Ion implantation demonstrates one of the best examples of successful path from the fundamental research to the high-level technology. The advantages of this technique in producing of a precise dose of impurity as well as uniform and shallow junction are indisputable [1]. This method found application in silicon-based device manufacturing, to form buried dielectric and metal layers, and in III–V technology also. At the same time, the bombardment of semiconductor with energetic ions inevitably produces the defect structure transformation that can improve or undesirably affect the device performance. For example, post-growth processing with cold, high fluence, Fe implantation was the key to produce InGaAsP-based THz devices with good emitter characteristics [2]. An ion implantation method for large-scale synthesis of high-quality graphene films [3, 4] and for InSbN layer formation at nitrogen incorporated into InSb wafer [5] was demonstrated. Another example deals with a low-energy implantation (25 keV) of thin (Ga,Mn)As layers with a very low fluence of either O or Ne ions completely suppressed ferromagnetism and which could be applied as a method for tailoring nanostructures in the layers [6].

One of the most important opportunities provided during implantation, in our opinion, is a wide spectrum of topological features induced on semiconductor surface by ion bombardment [7–10]. It was shown that normal-incidence ion beam can results in the formation of nanoscale objects on the surface of both elemental (Si, Ge [7, 8]) and compound semiconductor (GaSb [11, 12]). Well-ordered hexagonal arrays of InP nanodots [13] and well-aligned ripple structures on the surface of a single crystal of 3C-SiC [14] were created by oblique-incidence ion bombardment. Low-energy ion processing (from hundreds eV to tens of keV) creates peculiar surface morphologies, such as nano-ripples and nanodots, ranging from random to regular structures, whose electronic and optical properties are different from those of bulk materials and might find technological application for nanophotonics and nanoscale magnetism. For example, ion implantation is used to locally modify the solid surface to create periodic plasmonic microstructures with metal nanoparticles [15]. Thus, interest in developing technique for fabricating nanostructured semiconductor surfaces having varied textures and properties is increasing. However, we did not find any papers on the nanostructuring of ternary compounds induced by implantation.

As we know, HgCdTe (MCT) ternary compound is one of the basic semiconductors for photon detectors from NWIR to LWIR spectral range [16] that can absorb IR radiation over a broad range of wavelengths due to a change in the bandgap from 0 to 1.6 eV related to the change in the composition. The ability to detect sub-terahertz radiation by MCT-based structures is also discussed [17, 18]. Commonly used method for the fabrication of the IR devices based on MCT ternary compound is an ion implantation. An implant, getting into the epitaxial layer, initiates an active restructuring of the defect structure of MCT, which changes the epilayer carrier type. As a result, n-on-p (boron-implanted) [19] and p-on-n (arsenic-implanted) [20] photodiodes are fabricated. At the same time, it is well known that ion implantation induces mechanical stress in MCT layers, which is a matter of paramount importance for solid-state devices, and has been exploited to improve their electrical and optical properties. It was shown that implantation-induced stress is an important factor influencing the depth of p-n junctions in MCT-based structures [21].

This work aimed at studying the nanostructuring surfaces of a ternary chalcogenide semiconductor compound Hg_1 − *x*_Cd_*x*_Te (*x* = 0.223) performed using the processing with 100 keV B^+^ and Ag^+^ ion bombardment. We report on the studies of the evolution of surface morphology, the chemical compound and structural properties of the surface and subsurface region as well as charge transport of MCT epilayers subjected to the ion implantation. Considered here is the possibility to use well-known IR material which properties were changed under high-energy influence as detector for sub-THz radiation and, in such a way, to achieve operating range broadening. Role of the strain appearing upon implantation of ternary compound is discussed.

## Methods

Here, we have carried out a systematic study of mercury cadmium telluride thin films Hg_1 − *x*_Cd_*x*_Te (*x* ~ 0.223) which were grown on [111]-oriented semi-insulating Cd_1 − *y*_Zn_*y*_Te (*y* = 0.04) substrates from a Te-rich solution at 450 °C by liquid-phase epitaxy. The samples were irradiated by B^+^ and Ag^+^ ions on the side of the MCT epilayer (*d* = 17 μm) on “Vezuviy” implanter. The implantation energy and dose were 100 keV and *Q* = 3 × 10^13^ cm^−2^, respectively. Post-implantation thermal treatments were carried out under an Ar atmosphere at 75 °C for 5 h [22]. The temperature conditions and the technique of heat treatment of the samples allowed us to avoid the oxidation of the distorted layer (“to observe” a surface charge) and to activate ionic migration in the layer. All processed surfaces were examined after ion bombardment using atomic force (Digital Instruments NanoScope IIIa operating in the tapping mode) and a scanning electron microscope (MIRA3 TESCAN).

The structural characterization of the MCT samples was performed by X-ray diffraction (XRD) using a PANalytical X′Pert PRO triple-axis X-ray diffractometer. X-rays were generated from copper linear fine-focus X-ray tube. The CuK_*α*1_ line with a wavelength of 0.15418 nm was selected using a four-bounce (440) Ge monochromator. The experimental schemes allowed two cross sections of reciprocal lattice sites to be obtained: normally (*ω*-scanning) and in parallel (*ω*/2*θ*-scanning) to the diffraction vector. X-ray photoelectron spectroscopy (XPS) investigation was carried out using X-ray photoelectron spectrometer KRATOS equipped with a monochromatic Al*K*_*α*_ source.

Charge transport was investigated by the Hall Effect method and impedance spectroscopy. The concentration and mobility of carriers in MCT layers were determined from the Hall coefficient *R*_H_ and conductivity *σ* measurements which were made by the van der Pauw method in the magnetic field B of 0.01 up to 0.7 *T* at *T* = 80°K. High substrate resistivity excluded any influence of the one on results of electrical measurements. Samples 1 × 1 cm in size were cut for measurements from wafers.

To provide impedance spectroscopy characterization, a mesa-structure was prepared using the chemical etching of the sample in the standard etchant Br-HBr. Indium electrodes were deposited on faces of the sample. Impedance characteristics of the samples were studied using the precision impedance meter Z-3000X within the frequency range 1 Hz to 3 MHz with the amplitude of a sinusoidal signal 120 mV.

To obtain data about migration of impurity ions and intrinsic defects in the disordered area of MCT heteroepitaxial layer, the authors performed a model experiment by applying program packages TRIM_2008.

## Results

### Topological and Structural Characterization

It was found that the ion bombardment of the samples investigated has resulted in the essential change of the physical and structural properties of MCT surface. AFM and SEM methods permitted to find that in the range of nanoscale, arrays of holes and mounds are generated on a (111) MCT surface as a result of the normal incident ion bombardment. The histograms which present the superposition of the distribution functions of lateral dimensions in the *X*–*Y* plane were also constructed. The most probable size of nano-objects was determined as the position of the major maximum in the distribution histogram.

**Fig. 1:**
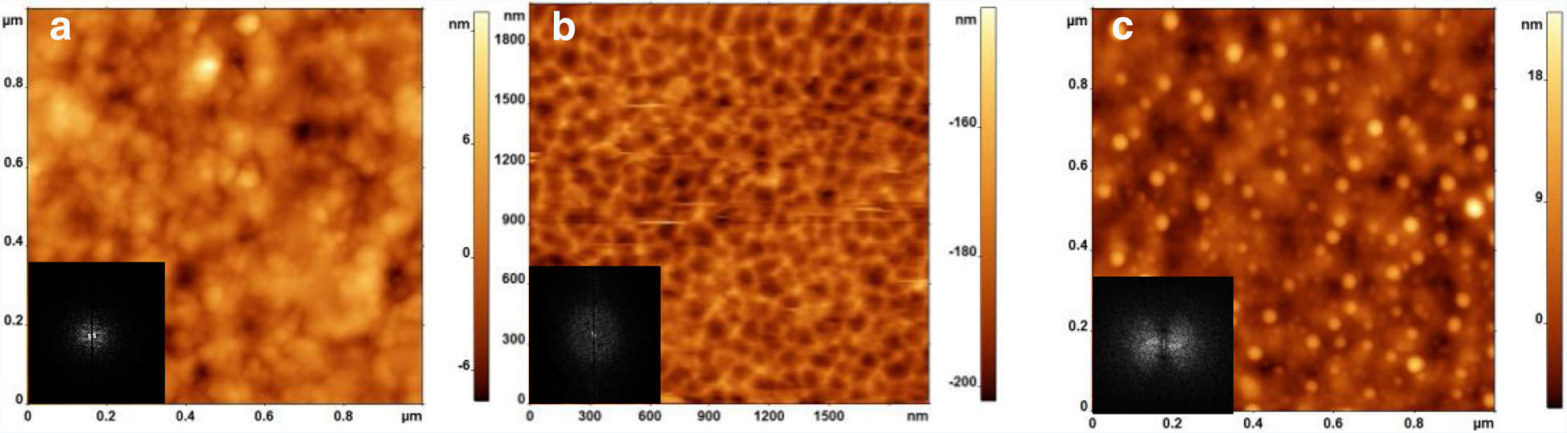
AFM images of a (111) MCT surface generated as a result of the ion bombardment with B^+^ and Ag^+^ ions. **a** Typical virgin surface. **b** B^+^ (*θ =* 0°, 100 keV, 3 × 10^13^ cm^−2^). **c** Ag^+^ (*θ =* 0°, 100 keV, 3 × 10^13^ cm^−2^). *Inset*: Fourier transforms of AFM images

Initial surface plane (see Fig. 1) is densely and regularly packed with round shaped grains with preferred size 25 nm in diameter. This means that the studied epitaxial film is characterized by a considerable nonequilibrium resource. As a rule, this state is concentrated in mechanical stresses of the local character (grains–pores), which is confirmed by the presence of a network of quasipores 3.5–10 nm in depth and 50–160 nm in diameter. The root-mean-square roughness (RMS) parameter for 1 × 1 μm^2^ initial surface fragments was in the energy range (2.45–3.34) nm.

The results of topometry based on AFM measurements show that in the range of nanoscale, arrays of holes and mounds are generated on a (111) MCT surface as a result of the normal incident ion bombardment. The results of electron microscopy confirmed these features in surface morphology of the treated samples. Figure 1b shows AFM reconstruction of periodic height modulations (“nanohole” pattern) induced on a MCT (111) surface with 100 keV B^+^ ion processing. After low-temperature annealing, MCT surface became denser—the study of the microhardness pointed to increase of its value to 12%. The ordered grid of quasipores is not observed. At the same time, some grains become consolidated. Silver ion bombardment (see Fig. 1c) gives rise to emergence of a uniform array of nano-islands 5 to 25 nm in height and with a base diameter of 13 to 35 nm. The corresponding 2D-fast Fourier transformation (FFT) has been depicted in insets of Fig. 1. They reveal that there is no signature of ordering of nanostructures over the surfaces for all regimes.

**Fig. 2:**
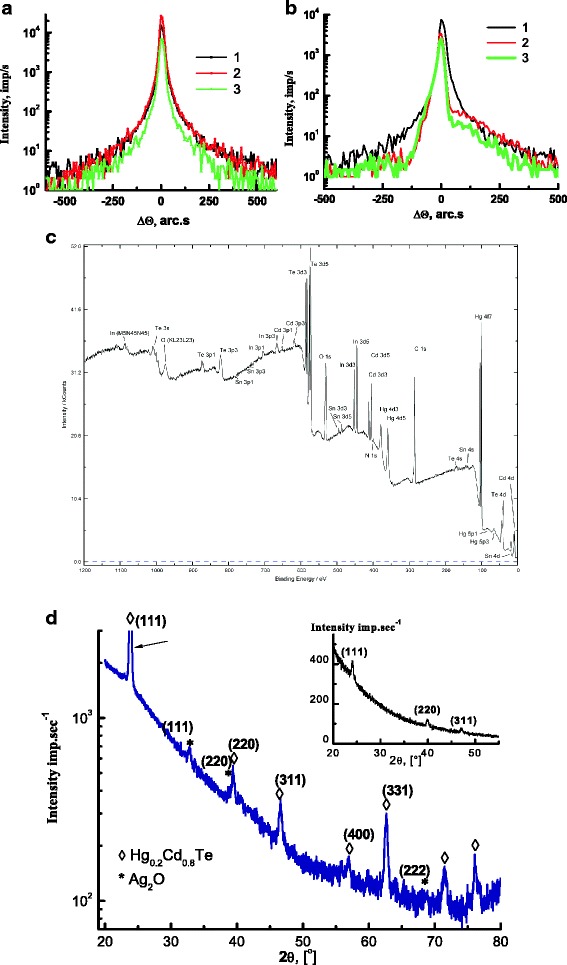
XRD and XPS characterization of typical samples investigated. **a** X-ray rocking curves for MCT-based structure: *1* initial, *2* boron implanted, and *3* annealed. **b** X-ray rocking curves for MCT-based structure: *1* initial, *2* silver implanted, and *3* annealed. **c** XPS survey spectrum of MCT-based structure after silver ion bombardment and annealing. **d** GI XRD spectrum of MCT-based structure after silver ion bombardment and annealing. *Inset* shows the X-ray diffraction spectrum of the sample in the Bragg configuration

Structural characterization of MCT surface before and after implantation was performed by XRD and XPS measurements. X-ray rocking curves (RC) for MCT-based structures were obtained from the symmetrical *ω*/2*θ* scanning. As seen in Fig. 2a, b (curve 1), the observed distribution of the intensity along axis *q*_*z*_ indicates the existence in the initial material of some structural heterogeneity caused by the existence of the vacancies (*q*_*z*_ < 0) and interstitials (*q*_*z*_ > 0). The micro-defect system in the initial material is apparently compensated that is confirmed by the symmetric form of the initial RC. The RCs for boron-implanted samples have symmetric form also whereas the RCs for silver-implanted samples have asymmetric form and are characterized by substantial shoulder on the high-angle side. XRD results in the coherent-scattering region point out to the compression of the boron-implanted MCT and tension of the silver-implanted MCT layers [23].

XPS measurements were performed to investigate the chemical state of MCT-based structures after ion bombardment. The survey XPS spectrum indicates that samples investigated are composed of Hg, Cd, and Te elements. O 1s peak at 531.0 eV, as well as In and Sn, was found in the MCT surface after silver bombardment (see Fig. 2c). At the same time, no peaks related to Ag were detected for sure. As we know, XPS method obtains information from within a few atomic layers of the surface and subsurface. Therefore, properties of the subsurface layer of the implanted MCT samples were studied by X-ray diffraction in the grazing-incidence (GI) scheme.

The GI diffractograms have been collected by irradiating the samples at an incident angle (*θ*_inc_) of 1°. The penetration depth was estimated by expression *2θ*_inc_ */μ*, where *μ* is the linear coefficient of X-ray attenuation that is ~1.5 × 10^3^ cm^–1^ for CdTe (and also CdZnTe) at the X-ray radiation energy used in [24]. Thus, GI XRD method in the current experimental conditions obtains information from ∼200 to 300 nm of subsurface region. Corresponding XRD spectrum of MCT-based structure is presented in Fig. 2d. It has confirmed the formation of the new phase in the subsurface region of MCT after silver implantation. This is MCT polycrystalline phase (ICDD PDF 00-051-1122). Besides, reflections attributable to cubic Ag_2_O (2*θ* = 32.8°, 38.2° according to ICDD PDF 00-041-1104) are appeared. It should be noted that the simulation of the implantation process performed in [25] using the TRIM2008 program package allowed us to state that the introduced impurity is mainly localized in the subsurface region (~100 nm) of MCT. The ellipsometry data also indicate the formation, in the silver-implanted CdHgTe/CdZnTe samples, of a distorted ~100-nm-thick layer with anomalous values of the extinction and refractory coefficients [25]. At last, data obtained by Transport of Ions in Matter simulation and ellipsometry for MCT samples after B^+^ bombardment point to the formation of an implantation-induced distorted layer ~400 nm in thickness.

Thus, from the results of AFM, XRD, and XPS measurements, it was found that in the range of nanoscale, arrays of holes and mounds on MCT (111) surface as well as the polycrystalline MCT cubic phase with alternative compound (*x* ~ 0.20) and a new phase of metal-oxide (Ag_2_O) have been fabricated using 100 keV ion beam irradiation of the basic material. Next, we examined the electrical properties of the samples investigated.

### Hall Measurements

The magnetic-field dependences of the Hall coefficient and the conductivity were measured. The change of the measurable parameters after implantation was observed for all samples. The Hall Effect data were processed in terms of the model, which includes several kinds of carriers using the next expression [26]:

$$ e{R}_H(B)=\frac{{\displaystyle \sum {a}_i{\mu}_i{c}_i}(B)}{{\left({\displaystyle \sum {c}_i(B)}\right)}^2+{B}^2{\left({\displaystyle \sum {a}_i{\mu}_i{c}_i(B)}\right)}^2} $$

where *e* is the electric charge, *c*_*i*_ *= n*_*i*_ *μ*_*i*_ */(1 + μ*_*i*_^*2*^ *B* ^*2*^ *)*, *n*_*i*_ is the concentration of the *i*th type of carrier, *μ*_*i*_ is the mobility of the *i*th type of carrier, *a*_*i*_ is the sign of carrier (−1 for electrons, +1 for holes), and *B* is the magnetic flux density. In addition, zero magnetic field electrical conductivity is given by *Σ*(0) *= eΣc*_*i*_(0)*.* From the analysis carried out, the electron and hole concentration and mobility were obtained before and after implantation (see Table 1).

**Table 1:** Some parameters of typically investigated MCT epilayers, *T* = 80 K

	Initial *p*-MCT epilayer, *x* = 0.223 *d* = 17 μm	*p*-MCT epilayer after Ag^+^ implantation, *x* = 0.207	Initial *n-*MCT epilayer, *x* = 0.223 *d* = 17 μm	*n-*MCT epilayer after B^+^ implantation, *x* = 0.223	*n-*MCT epilayer after Ag^+^ implantation, *x* = 0.2
Concentration, m^−3^	*p =* 3 × 10^22^ *n =* 10^15^ –	*p =* 7 × 10^21^ *n =* 3 × 10^18^ *p*_l_ *=* 10^17^	*n =* 4 × 10^21^ *n*_l_ *=* 5 × 10^18^	*n =* 6 × 10^22^ –	*n =* 1.5 × 10^22^ –
Mobility, m^2^ V^−1^c^−1^	*μ*_p_ *=* 0.01 *μ*_n_ *=* 8 –	*μ*_p_ *=* 0.012 *μ*_n_ *=* 0.8 *μ*_pl_ *=* 2.1	*μ*_n_ *=* 0.322 *μ*_nl_ *=* 8	*μ*_n_ *=* 0.1 –	*μ*_n_ *=* 0.3 –

It was revealed that initial samples were with *n-* and *p-*type of conductivity at 80 K. Initial dependences *R*_H_(*B*) and *σ*(*B*) for *p*-MCT epilayer were satisfactorily described by using combined electron and hole conductivity. The calculated values of the charge carrier parameters are presented in Table 1. After implantation, a tendency to the majority carrier (holes) concentration decrease and the electron concentration increase is observed. The mobility of majority carriers (holes) remains practically unchanged, while the electron mobility is decreased by an order of magnitude. Moreover, taking into account the light hole contribution was necessary. The character of the dependences *R*_H_(*B*) and *σ*(*B*) for *n*-MCT epilayer can be explained by the presence of electrons with high and low mobilities. After the implantation, the concentration of electrons with a low mobility increased from 4 × 10^21^ to 6 × 10^22^ m^−3^ in the boron-implanted specimens and to 1.5 × 10^22^ m^−3^ in the specimens implanted with silver ions. No contribution of high-mobility electrons was revealed for all *n*-type specimens after ion bombardment.

As it was mentioned in the previous paragraph, both TRIM simulation and ellipsometry results indicate the formation of an implantation-induced distorted layer with other properties in comparison with the basic material. This is evidenced by the X-ray data also. However, the Hall Effect data did not show multilayer formation, as it was obtained in our work devoted to the effect of high-frequency sonication on charge carrier transport in MCT [27]. At the same time, Hall Effect data simulation shows the composition reduction after Ag^+^ ion bombardment (see Table 1), which is agree with XRD results.

### Impedance Spectroscopy

Impedance spectroscopy is a very sensitive method for detection of non-stationary charge transport governed by charge-carrier relaxation in disordered semiconductors structure. Using the impedance technique, data equivalent to the real and imaginary parts of complex electrical values are measured as a function of the frequency of the applied electric field. The value and interpretation of impedance spectra are processed in analogy to equivalent circuits involving simple components such as resistors, capacitors, and inductors [28].

**Fig. 3:**
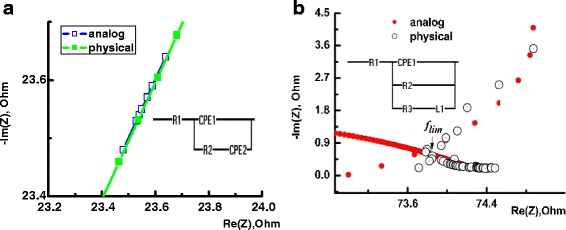
Impedance spectra (Nyquist plots) for MCT samples implanted with B^+^ (**a**) and Ag^+^ (**b**) ions. *Inset* shows the equivalent circuit model

The impedance measurements were performed at the G-Cp (parallel conductance and capacitance) configuration using Au plates as blocking electrodes. Figure 3a, b shows the complex impedance plane plots of the MCT samples implanted with B^+^ and Ag^+^ ions. Data for the boron-implanted sample are given for comparison. The arrow shows the direction of the increase in frequency. Symbols are the experimental data, and solid lines are the results of the fitting obtained using the EIS spectrum analyzer (http://www.abc.chemistry.bsu.by/vi/analyser/). Equivalent circuits obtained according to the Maxwell approach are shown in the insets of Fig. 3.

The impedance plane plot for the case of B^+^ implantation is in the shape of a line (Fig. 3a). On the equivalent circuits, R1 is the contact resistance. CPE is the constant phase element with impedance *Z*_CPE_ = *A* ^−1^(*iω*)^−*n*^ (*ω*—angular frequency), which is used to accommodate the nonideal behavior of the capacitance which may have its origin in the presence of more than one relaxation process with similar relaxation times [29]. The parameter *n* estimates the nonideal behavior having a value of zero for pure resistive behavior and is unity for capacitive behavior. For our case, CPE1 is a Warburg impedance element with *n =* 0.5, and CPE2 is a capacitance with *n =* 1. The series R2-CPE2 is a circuit which can be corresponding to the charge transport in the space charge region.

It is necessary to point out that the electrical circuit given in Fig. 3a is resembled with circuit presented in [30], which describes the behavior of an ideally polarized semiconductor that contains a reasonable concentration of inter-band defects. Feature consists in that instead of the capacitance, we have an element of Warburg, which manifests itself by a line in the low-frequency region and corresponds to mass transfer effect.

For the typical sample implanted by Ag^+^ ions, the hodographs of impedance is shown in Fig. 3b. At impedance locus, one can observe two clearly observed parts, namely, a ray followed by a small inductive loop. On the equivalent circuits, R1 is the contact resistance and R2-CPE1 is the resistors and capacitors in parallel combinations. It characterizes the conductivity and the charge of the disordered layer. The important peculiarity of its equivalent circuit is the presence of the reactive element inductance *L*, i.e., inductive-type impedance.

**Fig. 4:**
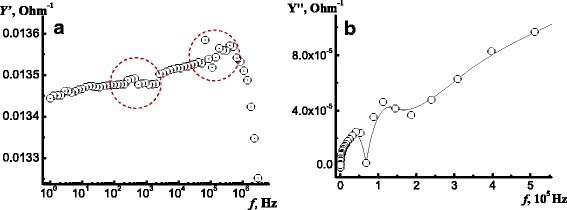
Bode plots for real (**a**) and imaginary (**b**) part of admittance of MCT sample implanted with Ag^+^ ions

Since components of the last equivalent circuits are electrically in parallel, it is convenient to consider admittance *Y* instead of impedance *Z*. Frequency dependence for real and imaginary part of admittance of a sample implanted by Ag^+^ ions on Fig. 4a, b was analyzed. From the analysis of the real part of admittance *Y′* (Fig. 4a), the frequency dispersion is not observed within the range (1 Hz…1 MHz). The imaginary part of admittance *Y′′* has practically very low value but within the total range of measurements increases by almost three orders of magnitude (Fig. 4b). In the low-frequency range (3 × 10^2^…3 × 10^3^) Hz, the silver-implanted samples show specific features that are indicative on resonance levels in structured material (first circle Fig. 4a). The observed sharp drop within the high-frequency range (1…3) MHz (second circle Fig. 4a) can be caused by geometrical and electrical relation of the studied samples.

## Discussion

Thus, we have demonstrated that the implantation resulted in semiconductor surface modification up to the nanoscale pattern formation. Furthermore, the examination of the electrical properties of MCT epilayers has confirmed the significant effect of the ion radiation treatment on both a charge and mass transfer phenomena in the material investigated.

A lot of experimental as well as theoretical efforts have been devoted to understand the mechanism of nanoscale structure formation on surfaces subjected to energetic ion bombardment. The widely accepted Bradley–Harper theory [31] explains pattern formation by the curvature dependence of the sputtering yield. An alternative approach is based on the theory (Cuerno [32] and Norris [33]) of the stress relaxation. In particular, Cuerno with co-authors show that nonuniform generation of stress across the damaged amorphous layer induced by their radiation is a key factor behind the range of experimental observations. We assume that the deformation fields appearing upon implantation of the studied heterostructure lead to the topological instability of the irradiated surface and are a determining factor of the observed surface transformation as well as of the change of the carrier transport.

The deformation sign is dependent on the ratio of ionic radii *r* ^+^ of the matrix atoms and introduced impurity [21]. Implantation with ions of small radius (such as B^+^, *r*_B_ ~ 0.97 Ǻ) stimulates the compression of the damaged layer, whereas the implantation with ions of radius comparable with that of Hg (in our case, these are Ag^+^ ions, *r*_Ag_ ~ 1.44 Ǻ, *r*_Hg_ ~ 1.55 Ǻ) gives rise to tensile stress in the damaged layer, as confirmed by the X-ray diffraction data obtained in this work and in our previous work [23, 34]. The calculated mechanical stress is *σ*_Ag_ = 2.2 × 10^5^ Pa (strain *ε* ~ 10^−6^) and *σ*_B_ = 1.4 × 10^3^ Pa (strain ε ~ 10^−8^) [25].

The structural transformation of the region subjected to the implantation is thought to occur due to the formation of a state with excess energy in a thin layer of the material. However, the accumulated energy is not sufficient for the formation of extended defects. We also did not observe the anisotropy of the surface morphology. While XRD studies in the coherent-scattering region point out to the implantation-induced deformation and post-annealing relaxation of MCT layers [23]. We need to emphasize that used implantation conditions (energy and fluence) are softer than those which stimulated formation of dislocation loops in MCT system [35]. An analysis of the initial sample surface indicates the substructural growth nonequilibrium of samples under study [34]. The charge particle (ion) flow additionally distorts the target lattice; in this case, its specific surface and the degree of disordering increase up to the formation of the distorted layer with the optical and electrical characteristics different than those observed for the matrix. The subsequent relaxation of the nonequilibrium state of the semiconducting material can pass via the formation of point defects in the crystal structure and the formation of new surface up to the excitation of solid-phase chemical reactions [36].

Additional factor affecting the surface morphology is the ion migration after implantation. Weak chemical bonds in material under study define the high concentration of electrically active intrinsic defects in accordance with the defect reaction Hg_*i*_ + V_Hg_ = Hg_Hg_ [21]. The migration of Hg_*i*_ was found to be the dominating process in MCT. The strain induced by the ion irradiation can shift this reaction to the left or to the right depending on the deformation sign and, in such a way, to make an impact on the parameters of the charge carriers. Defect migration in implanted MCT ternary compound is discussed in [25] in detail. Besides, applying strain on MCT removes the degeneracy at the Γ8 point by lowering the crystal symmetry. This can result in the decrease of the hole effective mass in the splitting subbands. In our experiment, the Hall Effect data point to the rise of the light-mass hole contribution in implanted samples. Thus, mechanical strain induced in ternary compound under high-energy influence is responsible to the evolution of MCT surface morphology as well as stipulates peculiarity of the mass and charge transport in this material.

At last, charge transport investigation with non-stationary impedance spectroscopy method has shown that boron-implanted MCT structures are characterized by capacity-type impedance whereas for silver-implanted MCT structures, an inductive-type impedance (or “negative capacitance”) is observed. It is known, that the inductive-type impedance (or “negative capacitance”) is observed in various semiconductor structures such as chalcogenide films, semi-insulating polycrystalline silicon, multilayer heterostructures, and metal-semiconductor interfaces; in homogeneous samples with an inertial–relaxational type of electrical conductivity; in bipolar transistors with insulating gate and Schottky diodes; and also in *p+–n* junction diodes fabricated on the basis of crystalline and amorphous semiconductor materials (see Ref. in [37]). It is believed that the disordered systems is characterized by the inductive-type impedance which caused by the processes of capture and retention of charge carriers at the trapping centers for a time [37, 38]. With regard to our case, a disordered layer with oxide inclusions (Ag_2_O) is induced by Ag^+^ ion bombardment and the trapping centers can be located at the oxide-semiconductor interface. The typical lifetime of carriers on these centers can be defined as *τ* ~ 1/2*f ~* (0.1–1) ms [37], where *f* was determined from the low-frequency peculiarity on the Bode plot for real part of admittance of MCT sample implanted with Ag^+^ ions (see Fig. 4a).

It should be also noted that the observed effect of ion beam nanostructuring as well as formation of the oxide inclusions (Ag_2_O) in semiconductor matrix can be useful from the viewpoint of developing a new class of electro-optical facility based on MCT that possesses a necessary combination of optical, electro-physical, and photoelectric properties. Because of the challenges put forward to miniaturizing modern detectors and communication devices, there is a problem in transferring power to and from small antennas, which could not break through the gain-bandwidth theory limit (Foster theorem) [39]. At that, circuits containing negative elements (“non-Foster” networks) are not constrained by gain-bandwidth theory and can achieve wide matching bandwidths with “difficult” loads arising from electrically short antennas.

We have assumed that it is possible to achieve operating range broadening in obtained MCT-based structure with inductive-type impedance. Really, the sensitivity of the hybrid structure, which integrates the nanostructured ternary compound (HgCdTe) with metal-oxide (Ag_2_O) inclusions, for sub-THz radiation was detected at 296 K. The millimeter (mm wave) source with ~140GHz frequency was used for testing of the MCT heterostructure responsivity that was found after the oblique-incidence (45°) Ag^+^ ion beam bombardment. The value of the measured signal was about 7–15 μV at output power ~7 mW. These measurements were performed using a lock-in detection scheme with modulation at 190 Hz. The signal was detected without amplification. NEP at *ν* ≈ 140 GHz and 296 K reaches 4.5 × 10^−8^ W/Hz^1/2^.

## Conclusions

Presented in this work are the results concerning of topological features of a semiconductor surface developed by the ion implantation. Modification of Hg_1 − *x*_Cd_*x*_Te-based structure was performed using the method of normal-incidence ion bombardment with boron and silver ions (100 keV), which was followed by low-temperature treatment. It was found that in the range of nanoscale, arrays of holes and mounds on Hg_1 − *x*_Cd_*x*_Te (111) surface as well as the polycrystalline Hg_1 − *x*_Cd_*x*_Te cubic phase with alternative compound (x ~ 0.20) and a new phase of metal-oxide (Ag_2_O) have been fabricated. Mechanical strain induced in MCT ternary compound under high-energy influence is responsible to the evolution of surface morphology as well as stipulates peculiarity of the mass and charge transport in this material. Charge transport investigation with non-stationary impedance spectroscopy method has shown that boron-implanted structures are characterized by capacity-type impedance whereas for silver-implanted structures, an inductive-type impedance (or “negative capacitance”) is observed. A hybrid system, which integrates the nanostructured ternary compound (HgCdTe) with metal-oxide (Ag_2_O) inclusions, was fabricated by Ag^+^ ion bombardment. The sensitivity of such metal-oxide-semiconductor hybrid structure for sub-THz radiation was detected with NEP ~ 4.5 × 10^−8^ W/Hz^1/2^at *ν* ≈ 140 GHz and 296 K without amplification.

## Abbreviations

AFM: Atomic force microscopy
CPE: Constant phase element
FFT: Fast Fourier transformation
GI: Grazing-incidence
IR: Infrared
LWIR: Long-wavelength infrared region
MCT: Mercury cadmium telluride
NWIR: Near-wavelength infrared region
RC: Rocking curves
RMS: Root-mean-square
SEM: Scanning electron microscope
TRIM: Transport of Ions in Matter
XPS: X-ray photoelectron spectroscopy
XRD: X-ray diffraction
